# Contrasting Effects of Extreme Drought and Snowmelt Patterns on Mountain Plants along an Elevation Gradient

**DOI:** 10.3389/fpls.2017.01478

**Published:** 2017-08-29

**Authors:** Sergey Rosbakh, Annette Leingärtner, Bernhard Hoiss, Jochen Krauss, Ingolf Steffan-Dewenter, Peter Poschlod

**Affiliations:** ^1^Chair of Ecology and Nature Conservation Biology, University of Regensburg Regensburg, Germany; ^2^Department of Animal Ecology and Tropical Biology, Biocenter, University of Würzburg Würzburg, Germany

**Keywords:** extreme climate event, drought, snowmelt, plant growth, sexual reproduction, plant trait, Alps

## Abstract

Despite the evidence that increased frequency and magnitude of extreme climate events (ECE) considerably affect plant performance, there is still a lack of knowledge about how these events affect mountain plant biodiversity and mountain ecosystem functioning. Here, we assessed the short-term (one vegetation period) effects of simulated ECEs [extreme drought (DR), advanced and delayed snowmelt (AD and DE), respectively] on the performance of 42 plant species occurring in the Bavarian Alps (Germany) along an elevational gradient of 600–2000 m a.s.l. in terms of vegetative growth and reproduction performance. We demonstrate that plant vegetative and generative traits respond differently to the simulated ECEs, but the nature and magnitude treatment effects strongly depend on study site location along the elevational gradient, species’ altitudinal origin and plant functional type (PFT) of the target species. For example, the negative effect of DR treatment on growth (e.g., lower growth rates and lower leaf nitrogen content) and reproduction (e.g., lower seed mass) was much stronger in upland sites, as compared to lowlands. Species’ response to the treatments also differed according to their altitudinal origin. Specifically, upland species responded negatively to extreme DR (e.g., lower growth rates and lower leaf carbon concentrations, smaller seed set), whereas performance of lowland species remained unaffected (e.g., stable seed set and seed size) or even positively responded (e.g., higher growth rates) to that treatment. Furthermore, we were able to detect some consistent differences in responses to the ECEs among three PFTs (forbs, graminoids, and legumes). For instance, vegetative growth and sexual reproduction of highly adaptable opportunistic graminoids positively responded to nearly all ECEs, likely on the costs of other, more conservative, forbs and legumes. Our results suggest that ECEs can significantly modify the performance of specific plant groups and therefore lead to changes in plant community structure and composition under ongoing climate change. Our study therefore underlines the need for more experimental studies on the effects of extreme climate events to understand the potential consequences of climate change for the alpine ecosystem.

## Introduction

Increased temporal climate variability caused by recent global warming has also inevitably led to an increased frequency and magnitude of extreme climate events (ECE; Meehl et al., 2000; IPCC, 2013; Trnka et al., 2014; Stott et al., 2016). More frequent extreme high and low precipitation and temperature events, such as heavy rainfall, summer drought (DR), as well as hot and cold spells, have already been reported in Central Europe (e.g., Yan et al., 2002; Schönwiese et al., 2003; Orth et al., 2016).

It has been proposed that the magnitude of these ECEs will be more intense at higher elevations (Trömel and Schönwiese, 2007; Rajczak et al., 2013) with severe impacts on alpine ecosystems and wildlife (Conedera et al., 2010; Jung et al., 2014; Chelli et al., 2016). For example, while high temperatures during one of the hottest European summer of 2003 resulted only in 9 and 5% shorter effective growing season length for colline and montane areas, respectively, the growing season was on average 12 and 64% longer in alpine and nival vegetation belts, respectively (Jolly et al., 2005). However, despite the fact that the ECEs have a greater influence on ecosystems and societies than gradual shifts and trends of mean temperatures and the precipitation regime (Jentsch et al., 2007; Beier et al., 2012), there is a substantial lack of knowledge regarding how these events affect mountain plant biodiversity and mountain ecosystem functioning (Leingärtner et al., 2014; Hoiss et al., 2015; Wellstein et al., 2017).

Only in the latest decade, several studies on the effects of ECEs on mountain vegetation have been carried out (Bigler et al., 2007; Jung et al., 2014; Orsenigo et al., 2015), with a strong focus placed on the consequences of changes in winter precipitation (Wipf and Rixen, 2010; Livensperger et al., 2016). Snow cover determines plant life in mountain regions, because the thermal capacity of snow effectively buffers soil and plant temperatures during the cold season, thus protecting plants from freezing (Sakai and Larcher, 1987; Kreyling, 2010). It also affects the water economy of soils and plants in spring through melting snow (Gerdol et al., 2013). Furthermore, snow cover can ameliorate soil nutrient status because of an increased nutrient influx from wind-blown litter and/or higher activity of soil microbes releasing nutrients at higher concentrations during snowmelt (Yano et al., 2015). Therefore, changes in snow cover thickness and duration caused by the ECEs may also influence the timing and duration of phenological events (Cornelius et al., 2013; Petraglia et al., 2014), as well as vegetative growth and reproductive efforts of mountain species (Gerdol et al., 2013; Wheeler et al., 2016). For instance, a prolongation of snow cover duration often delays plant phenology (Inouye, 2008; Cooper et al., 2011), whereas an earlier snowmelt can advance reproductive phenology (Dunne et al., 2003; Wipf and Rixen, 2010; Cornelius et al., 2013; Dorji et al., 2013). These responses were also found to be highly specific to species and functional type (Cornelius et al., 2013; Bollig and Feller, 2014; Wellstein et al., 2017). However, the elevational differences in plant response to manipulations of snow cover have been rarely investigated, despite the fact that these responses may be particularly stronger at higher elevations, where snow pack duration is one of the main limiting factors for plant growth and development (Körner, 1999; Ellenberg and Leuschner, 2010).

Although the plant responses to manipulations of snowpack thickness and duration are widely reported [see for example a review from Wipf and Rixen (2010)], studies on the effects of extreme drought events on mountain plant performance are generally underrepresented (however, see Bollig and Feller, 2014; Jung et al., 2014; Chelli et al., 2016). From experiments carried out in lowland conditions it is known that drought can limit plant photosynthetic capacity and therefore growth by decreasing soil moisture (Kreyling et al., 2008; Reyer et al., 2013; Wellstein et al., 2017). The severity of drought effects on mountain plant water status is supposed to increase with altitude in humid temperate mountains such as the European Alps, due to the strong dependence of upland species on relatively high precipitation and high soil moisture of high altitudes as compared to lowlands (Körner, 1999; Gitlin et al., 2006; Ellenberg and Leuschner, 2010; Garcia-Fernandez et al., 2013). Furthermore, in some nutrient-limited ecosystems, such as mountains, drought also can limit plant growth capacity by reducing soil microbial activity (Sardans et al., 2008).

In this study, we focus on the short-term (one vegetation period) response of 42 plant species occurring in the Bavarian Alps (Germany) along an elevational gradient of 600–2000 m a.s.l. to three particular ECEs – extreme drought, advanced (AD), and delayed (DE) snowmelt – in terms of vegetative growth and reproduction performance. Our first aim was to study the overall treatment effects on the mountain species’ performance. Secondly, we focused on the magnitude of the treatment along the elevational gradient. In particular, we expected that effects of the snow manipulation experiments would be stronger at higher altitudes, because the length of the vegetation period in upland strongly depends on the duration of snowpack and consequently the time of the snowmelt. In addition, we expected that extreme drought would also have a stronger negative impact on plant performance in the uplands, because under natural conditions of higher altitudes the probability of extreme drought is relatively low, due to higher precipitation and air humidity (Körner, 1999; Gitlin et al., 2006; Ellenberg and Leuschner, 2010; Garcia-Fernandez et al., 2013).

Next, we asked whether the species’ response to the treatments differed according to their altitudinal origin. Any mountain flora consists of species with different altitudinal origins, which have evolved in response to their particular altitudinal environment. We therefore assumed that upland species would be more sensitive to extreme drought when compared to their lowland counterparts. As for the snow manipulation experiment, we expected that both lowland and upland species would improve their performance with the advanced snowmelt (AD), because any process that enhances the growing season in a mountain environment will maximize plant performance (Woodward et al., 1986; Sardans et al., 2008; Wipf and Rixen, 2010). However, the positive response of the lowland species to the AD should be stronger, because plants in this group possess the inherent ability to increase growth rates as daily air temperatures increase (Woodward, 1979). Consequently, an opposite pattern is expected in the case of the delayed snowmelt (DE).

Finally, we searched for a possible consistent response of functional groups, in order to delimit the complexity of a single plant’s responses to the treatments. In order to generalize the observed plant responses to the ECEs, the concept of plant functional type (PFT) was applied to our study, with a PFT defined as a grouping of species sharing aboveground and belowground morphology, physiology, phenology and competitive ability (Wellstein et al., 2017 and citations therein) making different responses to ECE likely (e.g., Dormann and Woodin, 2002; Parmesan and Hanley, 2015).

## Materials and Methods

### Study Region and Site Selection

Fieldwork was carried out in the Berchtesgaden National Park located in the Bavarian Alps (south-east Germany). The National Park is approx. 200 km^2^ in area and characterized as typically alpine topography, with steep mountain peaks composed of Triassic limestone and dolomite (Marke et al., 2013). The climate is typically montane with large altitudinal decrease in mean annual air temperatures from +7 to -2°C from 603 to 2713 m above sea level (a.s.l.), respectively. Mean annual precipitation in the region varies, ranging from approximately 1500 to 2600 mm (Marke et al., 2013).

Between February 20th and April 15th, 2010, 15 grasslands were chosen in the study region along an elevational gradient (600–2000 m a.s.l; Appendix 1). All selected grasslands belong to a single vegetation type (calcareous grasslands) and share similar habitat characteristics with regard to aspect, slope, presence of rocky outcrops and intensity of land use (all sites are either not managed or slightly grazed); thus the experimental treatments are likely to be the main explanatory variable for the target plant species’ responses. In order to exclude the effect of grazing animals (cattle, sheep, or wild deer) on plant performance, several study sites were enclosed by electric fences (Appendix 1). We did not observe any impact of the grazer on plant performance in the unfenced study sites.

### Experimental Treatments

**FIGURE 1:**
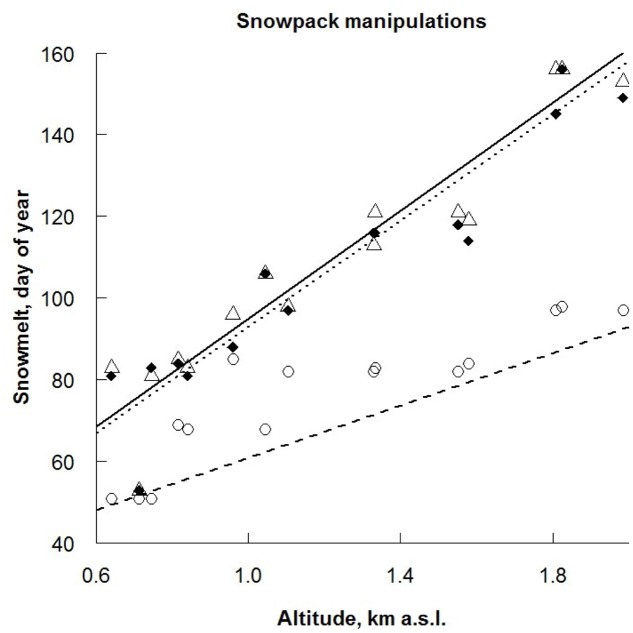
Final day of snowmelt for three treatments: ● control (CO) (solid line), ∘ advanced snowmelt (AD) (dashed line) and Δ delayed snowmelt (DE) (dotted line) in 15 study sites in relation to altitude. The differences in intercepts and slopes of the regression lines are based on estimated parameters for linear model (*r*^2^ = 0.89, *F* = 64, df = 42, *p* < 0.001).

In each site, four experimental treatments were carried out, all in 4 m × 4 m plots, namely: advanced and delayed snowmelt (AD and DE, respectively), extreme drought (DR), and control (CO). In order to simulate the advanced and delayed snowmelt, snow was shoveled from the AD plots to the DE plots until only a thin layer was left on the former. The removal of snow meant there was an advanced snowmelt of 28 ± 4 days, which increased with the altitude, while the added layer of snow lead to a delayed melt of 2 ± 4 days (**Figure 1** and Appendix 1). Four weeks after the snow had melted (the date of snowmelt is altitude specific; Appendix 1), we simulated an extreme drought event by constructing 4 m × 4 m rain-out shelters with aluminum tubes and cast-iron key clamps (B-One key clamps, Montfoort, The Netherlands) and covered them with a transparent plastic sheet (0.2 mm polyethylene, SPR 5, Hermann Meyer KG, Germany), which allowed nearly 90% penetration of photosynthetically active radiation. The rain-out shelters had a roof height of 125 cm at the highest point and had two open sides to avoid greenhouse effects, allow air circulation and pollinators access to the flowering plants. Intensity of the DR treatment was based on the local 1000-year extreme event (Jentsch and Beierkuhnlein, 2008; Jentsch et al., 2009). Due to the lack of long-term meteorological observations in high altitudes (above 1000 m a.s.l.) of the study region, vegetation periods (March–September) of 1961–2000 for lowland experimental sites (600–900 m a.s.l.) were used as the reference period (data: German Weather Service). Gumbel I distributions were fitted to the annual extremes, and 1000-year recurrence events were calculated (Gumbel, 1958). Extreme drought was defined as the number of consecutive days with <1 mm daily precipitation. Accordingly, a drought period of 43 days was applied during the first half of the growing season between 05th May to 25th July, depending on altitude (Appendix 1). The fourth plot was designated control and remained unmanipulated.

Soil surface temperatures were measured with temperature loggers (Thermochron iButtons DS1921G#F5; Maxim Integrated Products, Inc., Sunnyvale, CA, United States) every 2 h, and when the surface air temperature reached over +5°C for three consecutive days the plots were said to be snow free (Wipf et al., 2006). Additionally, we measured soil surface temperature in the DR treatment (every 2 h, 43 days long), in order to control for undesirable warming effect on the treatment on plant performance (Appendix 1). Mean soil surface air temperature under the rain-out shelters was 14.5 ± 2.1°C during the drought period and 14.4 ± 2.2°C on the control plots, thus no significant differences between rain-out shelters and control plots existed (paired *t*-test: *t*_12_ = -0.5, *P* = 0.6).

**FIGURE 2:**
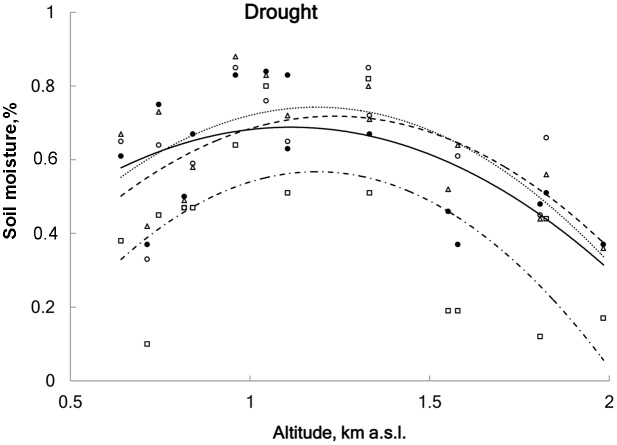
Averaged soil moisture content for four treatments: ● control (solid line), ∘ advanced snowmelt (dashed line), Δ delayed snowmelt (dotted line) and □ drought (DR) (dot-dashed line) in 15 study sites in relation to altitude. DR treatment significantly uniformly reduced soil moisture content (on average 17% less as compared to control), whereas advanced and delayed snowmelts did not affect soil moisture content (linear regression; *r*^2^ = 0.43, *F* = 11.2, df = 59, *p* < 0.001).

Soil moisture was measured weekly using a portable soil moisture meter at 60 mm depth (Delta-T Devices type HH2+ ThetaProbe ML2x sensors, Cambridge, United Kingdom). When averaged over all sites, soil moisture was significantly reduced in DR plots (63%) compared to the CO plots (80%; linear regression, *r*^2^ = 0.43, *F* = 11.2, df = 59, *p* < 0.001); no significant difference in soil moisture was found among control, advanced and delayed snowmelt (**Figure 2** and Appendix 1). Additionally, we installed rain collectors to measure the amount of rain that was excluded from the DR treatment. Mean rainfall over all study sites during the drought period was 379 ± 71 l/m^2^ and the amount of rain did not show a directional change along the elevational gradient (linear regression, *r*^2^ = 0.03, *F* = 1.1, df = 14, *p* = 0.3).

More detailed discussion on the experimental design and treatments is shown below (Cornelius et al., 2013; Leingärtner et al., 2014; Hoiss et al., 2015).

### Trait Selection and Measurement

#### Vegetative Traits

The effects of the ECEs on vegetative growth were estimated by measuring such leaf traits as specific leaf area (SLA), as well as leaf carbon and nitrogen contents (LCC and LNC, respectively). Previous studies have identified that SLA is positively correlated with photosynthetic rates on a leaf-mass basis and therefore relative growth rates (Cornelissen et al., 2003; Kleyer et al., 2008). Variation in SLA values has been connected to climatic variation, where heat, cold and drought stress all tend to select for leaves with relatively small SLA values (Reich et al., 1999; Woods et al., 2003; Kleyer et al., 2008). Additionally, low soil nutrient content reduces SLA (Cunningham et al., 1999). LCC is positively correlated to growth rates as well as SLA; temperature increases usually enhance the photosynthetic capacity that leads to increased concentrations of photosynthetics in leaves and/or their allocation to new tissue construction (Tolvanen and Henry, 2001). Increased concentrations of leaf carbon may also indicate an accumulation of osmoprotectors in the leaves, as a reaction to drought (Sardans et al., 2008). LNC is integral to the proteins of photosynthetic machinery (especially Rubisco) and structural proteins of plant cells (Poorter and Bergkotte, 1992). Due to its negative correlation with relative growth rate, the trait values are found to increase with decreasing ambient temperatures or a shortening of the growing period (Körner et al., 1986; Wright et al., 2004). Furthermore, LNC reflects the effects of drought on biomass accumulating by changing nitrogen soil availability (Sardans et al., 2008). In addition, this trait can provide information on how plants utilize mineral nutrients available in soil (Bowman et al., 1995; Körner, 1999).

Only species with a presence of at least ten individuals in at least two experimental plots, including the control plot, have been used for further trait measurements. This resulted in the 42 species (Appendix 2).

For the measurements of SLA, LCC, and LNC, three fully expanded non-damaged sun leaves with petioles were collected from each of five randomly selected full flowering individuals per species and treatment. The leaves were collected in all treatment plots on the same day when the rain-out shelters were removed from the DR treatment plots (from 05th May – to 25th July, depending on altitude; Appendix 1). All of the collected specimens were located in the central (2 m × 2 m) part of the plot in order to avoid undesirable margin effects. Measurements of SLA, LCC, and LNC followed standardized commonly used protocols (Perez-Harguindeguy et al., 2013).

#### Generative Traits

In the present study, we focused on traits that quantify plant reproduction performance, which were seed number per ramet and seed mass (Kleyer et al., 2008). The relevance of these traits for studies on plant-climate interaction has been previously confirmed in experimental studies regarding the effects of manipulated climates on plant sexual reproduction (e.g., Stenström et al., 1997; Totland and Alatalo, 2002).

The reproductive traits were measured in all plots at the time when the majority of the target species in the plot had ripe seeds (from beginning of July to mid of September, depending on altitude). We collected all the seeds from 10 individuals of a species per treatment in paper bags, air-dried them, removed flower remnants and processed them. Seed number is given as the number of seeds (or caryopses in case of graminoids) per individual. In order to obtain seed mass, we randomly selected from 20 to 100 (depending on sample size) seeds and subsequently weighed them.

### Statistical Analysis

All statistical calculations were performed in R software version 3.2.0 (R Core Development Team, 2016).

To assess the effects of the treatments on the plant traits, we calculated linear models for a trait as the response variable and treatment as explanatory variable. The statistical analysis was carried out in three steps. First, we tested for overall treatment effects on each trait separately. Additionally, an interaction (Treatment^∗^Elevation) was included in the model, in order to estimate differences in a treatment’s magnitude of effect along the elevational gradient. Second, we tested the effects of the treatments on species of different altitudinal origin, by including in the model a two-level factor (‘lowland’ and ‘upland’; Appendix 2). To classify the study plant species according to their altitudinal origin, we surveyed vegetation at 40 sites along an altitudinal gradient from 650 to 2570 m a.s.l. and determined distributional (and therefore climatic) ranges of our target species (Rosbakh et al., 2015). Ten random 1-m^2^ plots were set up at each site and in each plot we recorded the cover of each vascular plant species. The relative abundance of a species at a site was calculated as the mean value (% cover) of its abundance in all plots. If the species had the highest relative abundance in subalpine or/and alpine vegetation belt (in our study system, 1400–1700 and 1800–2500 m a.s.l.), then it was classified as ‘upland.’ The remaining species with the highest relative abundances in mountain vegetation belt, i.e., below 1400 m a.s.l. were placed into ‘lowland’ category. To estimate the trait response under the experimental treatments along the altitudinal gradient, an interaction Altitudinal origin^∗^Treatment^∗^Elevation was also included in the model as an explanatory variable.

In the third stage, we tested for differences in trait responses to the experimental treatments among three most frequent in European grasslands functional groups, forbs, graminoids and legumes (see respective columns in Appendix 2), by including functional groups in the model as interactions with treatment. Previous studies have demonstrated that these particular PFTs differ in photosynthetic rates, nitrogen and water-use efficiencies, tissue turnover, plasticity of resource allocation, the rate of growth response following changes in resource supply and stem and root morphology, such as clonality or type of root system (Bowman et al., 1995; Zhang and Welker, 1996; Zavaleta et al., 2003; Klanderud, 2008). These parameters, therefore, may determine their sensitivity toward the ECEs. The effect of altitude on the trait response to the treatments (Functional group^∗^Treatment^∗^Elevation) was estimated as well.

In order to meet the preconditions of the linear models (i.e., normal distribution of the residuals and homogeneity of variances), values of some traits were either log- or square root-transformed, if necessary.

For the sake of brevity, several regression models were set up to illustrate the treatment effect on each trait, based on significant differences in slopes and intercepts.

## Results

### Trait Responses to the Treatments

**FIGURE 3:**
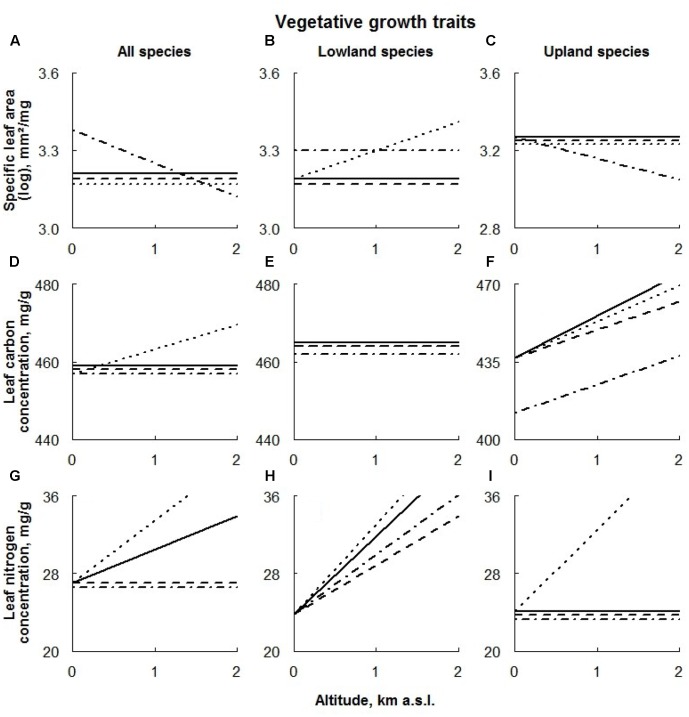
Visual description of the linear models showing the effects of extreme climate events (ECE) on vegetative growth traits (**Table 1**) averaged over all species (‘All species’; **A,D,G**) and species of different altitudinal origin [‘Altitudinal origin’ (lowland: **B,E,H**; upland: **C,F,I**)]. Solid line: control treatment; dashed line: AD treatment; dotted line: DE treatment; dot-dashed line: DR treatment (for details see Materials and Methods section).

**FIGURE 4:**
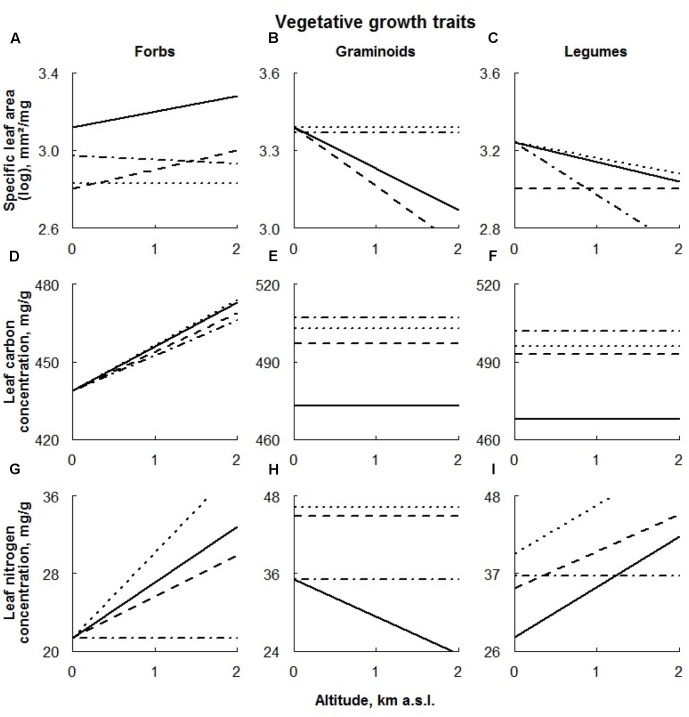
Visual description of the linear models showing the effects of ECE on vegetative growth traits of the different plant functional types [‘Forbs’ **(A,D,G)**, ‘Graminoids’ **(B,E,H)**, and ‘Legumes’ **(C,F,I)**; **Table 2**]. Solid line: control; dashed line: AD treatment; dotted line: DE treatment; dot-dashed line: DR treatment (for details see Materials and Methods section).

**FIGURE 5:**
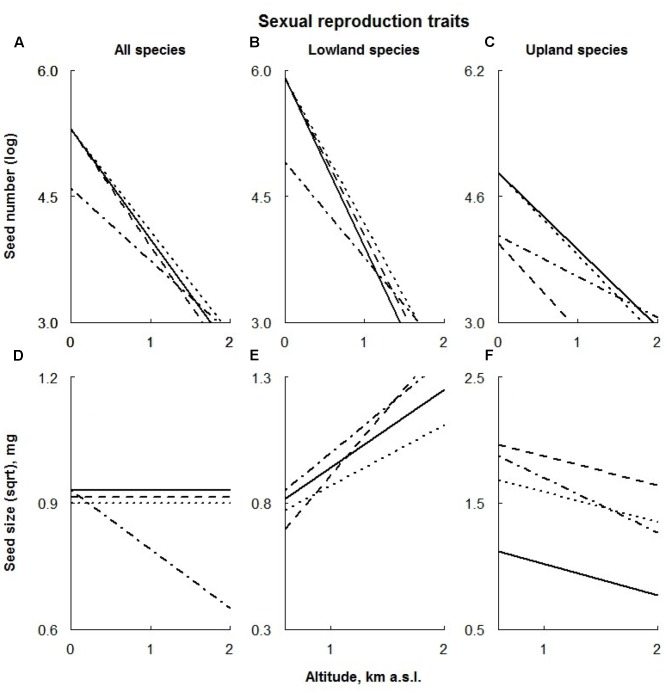
Visual description of the linear models showing the effects of ECE on reproductive traits (**Table 1**) averaged over all species (‘All species’; **A,D**) and species of different altitudinal origin [‘Altitudinal origin’ (lowland: **B,E**; upland; **C,F**)]. Solid line: control; dashed line: AD treatment; dotted line: DE treatment; dot-dashed line: DR treatment (for details see Materials and Methods section).

**FIGURE 6:**
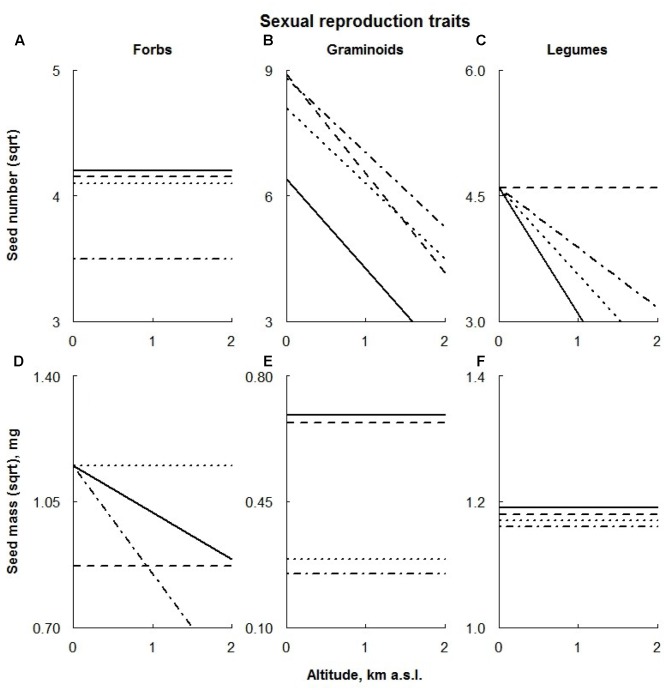
Visual description of the linear models showing the effects of ECE on reproductive traits of the different PFTs [‘Forbs’ **(A,D)**, ‘Graminoids’ **(B,E)**, ‘Legumes’ **(C,F)**; **Table 2**]. Solid line: control; dashed line: AD treatment; dotted line: DE treatment; dot-dashed line: DR treatment (for details see Materials and Methods section).

Vegetative growth and seed reproduction traits showed significant responses to the experimental ECE (**Figures 3–6** and **Tables 1**, **2**).

**Table 1:** Estimated intercepts (*B*; trait value at 0 m a.s.l.) and slopes (*m*; change in trait unit per km) for linear models and their significance showing effects of extreme climate events (ECE) on five studied functional traits averaged over all species (‘All species’) and species of different altitudinal origin (‘Altitudinal origin’).

				Altitudinal origin			
				
		All species		Lowland		Upland	
				
Trait	Treatment	*B*	*m*	*B*	*m*	*B*	*m*
**Specific leaf area (log), mm^2^/mg^-1^**	Control	3.20 ± 0.10^∗∗∗^	0.01 ± 0.08	3.14 ± 0.11^∗∗∗^	0.05 ± 0.05	3.43 ± 0.05^∗∗∗^	-0.13 ± 0.09
	Advanced	3.18 ± 0.03	0.04 ± 0.08	3.13 ± 0.04	0.13 ± 0.05	3.63 ± 0.05^∗∗∗^	-0.18 ± 0.09
	Delayed	3.21 ± 0.03	0.05 ± 0.08	3.10 ± 0.04	0.20 ± 0.05	3.70 ± 0.05^∗∗∗^	-0.19 ± 0.09
	Drought	3.37 ± 0.03^∗∗∗^	-0.11 ± 0.08^∗^	3.23 ± 0.04^∗^	0.08 ± 0.05	3.68 ± 0.05^∗∗∗^	-0.33 ± 0.09^∗^
**Leaf carbon concentration, mg/g**	Control	461 ± 6^∗∗∗^	3.58 ± 4.46	466 ± 6^∗∗∗^	-0.58 ± 5.29	442 ± 9^∗∗^	15.79 ± 6.40^∗^
	Advanced	461 ± 5	4.61 ± 4.52	465 ± 6	0.52 ± 5.32	425 ± 10	12.93 ± 6.84
	Delayed	458 ± 5	6.48 ± 4.49	462 ± 6	3.18 ± 5.24	425 ± 10	14.84 ± 6.60^∗^
	Drought	465 ± 5	0.27 ± 4.56	470 ± 7	-4.17 ± 5.64	419 ± 10^∗^	11.45 ± 6.62
**Leaf nitrogen concentration, mg/g**	Control	26.7 ± 2.6^∗∗∗^	4.2 ± 2.1	23.9 ± 2.8^∗∗∗^	8.2 ± 2.3^∗∗∗^	23.9 ± 3.6	4.2 ± 2.7
	Advanced	27.6 ± 2.2	3.2 ± 2.1	26.5 ± 2.5	5.2 ± 2.3^∗^	18.7 ± 3.9	5.9 ± 2.9^∗^
	Delayed	24.2 ± 2.2	6.7 ± 2.1^∗∗^	22.8 ± 2.5	9.3 ± 2.3^∗∗∗^	19.9 ± 3.8	8.5 ± 2.8^∗∗^
	Drought	31.1 ± 2.2^∗^	-0.4 ± 2.1	25.5 ± 2.6	6.9 ± 2.4^∗∗^	27.4 ± 3.9	-1.7 ± 2.8
**Seed number (log)**	Control	5.2 ± 0.4^∗∗∗^	-1.3 ± 0.4^∗∗^	6.0 ± 0.5^∗∗∗^	-2.1 ± 0.4^∗∗∗^	4.8 ± 0.4^∗∗^	-0.9 ± 0.4^∗^
	Advanced	5.3 ± 0.2	-1.4 ± 0.4^∗∗^	5.8 ± 0.3	-2.0 ± 0.4^∗∗∗^	4.0 ± 0.4	-1.2 ± 0.4^∗^
	Delayed	5.0 ± 0.2	-1.2 ± 0.4^∗∗^	5.6 ± 0.3	-1.8 ± 0.4^∗∗∗^	4.1 ± 0.4	-1.0 ± 0.4 ^∗^
	Drought	4.6 ± 0.2^∗∗∗^	-0.8 ± 0.4^∗^	5.0 ± 0.3^∗∗∗^	-1.3 ± 0.4^∗∗^	3.8 ± 0.4^∗∗^	-0.5 ± 0.4
**Seed mass (sqrt), mg**	Control	1.0 ± 0.2^∗∗∗^	-0.03 ± 0.17	0.7 ± 0.2^∗∗^	0.3 ± 0.2	1.4 ± 0.1^∗∗∗^	-0.3 ± 0.2
	Advanced	0.9 ± 0.1	0.05 ± 0.17	0.5 ± 0.1^∗^	0.5 ± 0.2^∗∗^	2.2 ± 0.1^∗∗∗^	-0.2 ± 0.2
	Delayed	1.0 ± 0.1	-0.03 ± 0.17	0.7 ± 0.1	0.3 ± 0.2	2.1 ± 0.1^∗∗∗^	-0.3 ± 0.2
	Drought	1.1 ± 0.1^∗^	-0.16 ± 0.17	0.6 ± 0.1	0.4 ± 0.2^∗^	2.4 ± 0.1^∗∗∗^	-0.5 ± 0.2^∗∗^


*To visualize the results of the linear models, several regression lines for each treatment and each trait studied were drawn, based on significance for slopes and intercepts (**Figures 3**, **5**). Significance levels: ^∗∗∗^*p* < 0.001, ^∗∗^0.001 < *p* < 0.01, ^∗^0.01 < *p* < 0.05.*

**Table 2:** Estimated intercepts (*B*; trait value at 0 m a.s.l.) and slopes (*m*; change in trait unit per km) for linear models and their significance showing effects of ECE on five studied functional traits of the different PFTs (‘Forbs,’ ‘Graminoids,’ ‘Legumes’).

		Forbs		Graminoids		Legumes	
				
Trait	Treatment	*B*	*m*	*B*	*m*	*B*	*m*
**Specific leaf area (log), mm^2^/mg^-1^**	Control	3.10 ± 0.06^∗∗^	0.11 ± 0.08	3.29 ± 0.12^∗∗∗^	-0.03 ± 0.10	3.25 ± 0.06	-0.09 ± 0.09
	Advanced	2.86 ± 0.06^∗∗∗^	0.12 ± 0.08	3.34 ± 0.07	-0.09 ± 0.10	3.12 ± 0.07^∗^	-0.03 ± 0.09
	Delayed	2.89 ± 0.06^∗∗∗^	0.17 ± 0.08	3.27 ± 0.07	0.06 ± 0.10	3.29 ± 0.07	-0.09 ± 0.09
	Drought	2.93 ± 0.07^∗∗^	0.00 ± 0.08	3.42 ± 0.08	-0.10 ± 0.1	3.30 ± 0.08	-0.26 ± 0.06^∗∗^
**Leaf carbon concentration, mg/g**	Control	438 ± 7^∗∗∗^	18.0 ± 5.0^∗∗∗^	483 ± 9^∗∗∗^	-11.7 ± 9.1	471 ± 8^∗∗∗^	-11.7 ± 5.8
	Advanced	441 ± 7	17.2 ± 5.2^∗∗^	511 ± 10^∗∗^	4.9 ± 9.1	502 ± 8^∗∗∗^	4.9 ± 5.9
	Delayed	437 ± 7	20.0 ± 5.0^∗∗∗^	519 ± 9^∗∗∗^	2.2 ± 8.5	503 ± 8^∗∗∗^	2.2 ± 6.0
	Drought	441 ± 7	15.1 ± 5.1^∗∗^	523 ± 10^∗∗∗^	-6.0 ± 9.8	507 ± 8^∗∗∗^	-6.0 ± 6.4
**Leaf nitrogen concentration, mg/g**	Control	21.9 ± 2.9^∗∗∗^	5.1 ± 2.2^∗^	30.0 ± 3.5^∗^	0.6 ± 3.5	28.8 ± 2.8^∗^	7 ± 2.5^∗∗^
	Advanced	22.3 ± 2.5	4.3 ± 2.2	36.2 ± 3.5	3.1 ± 3.5	37.0 ± 2.8^∗∗^	5.1 ± 2.5^∗^
	Delayed	17.9 ± 2.5	8.6 ± 2.2^∗∗∗^	38.6 ± 3.3^∗^	5.7 ± 3.4	41.2 ± 2.9^∗∗∗^	6.6 ± 2.5^∗^
	Drought	26.0 ± 2.5	0.1 ± 2.2	31.0 ± 3.6	4.9 ± 3.8	37.1 ± 3.0^∗∗^	1.8 ± 2.7
**Seed number (log)**	Control	4.1 ± 0.3^∗∗∗^	-0.1 ± 0.3	6.3 ± 0.2^∗∗∗^	-2.1 ± 0.3^∗∗∗^	5.3 ± 0.4^∗∗^	-2.0 ± 0.4^∗∗∗^
	Advanced	4.0 ± 0.3	-0.1 ± 0.3	8.8 ± 0.3^∗∗∗^	-2.3 ± 0.3^∗∗∗^	5.2 ± 0.6	-1.0 ± 0.5^∗^
	Delayed	4.1 ± 0.2	-0.2 ± 0.3	8.0 ± 0.3^∗∗∗^	-1.7 ± 0.3^∗∗∗^	6.0 ± 0.5	-1.5 ± 0.4^∗∗∗^
	Drought	3.4 ± 0.2^∗∗^	0.3 ± 0.3	8.7 ± 0.2^∗∗∗^	-1.7 ± 0.3^∗∗∗^	6.5 ± 0.5^∗∗^	-1.2 ± 0.4^∗∗^
**Seed mass (sqrt), mg**	Control	1.3 ± 0.2^∗∗∗^	-0.25 ± 0.18	0.6 ± 0.1^∗∗∗^	0.20 ± 0.18	1.4 ± 0.1	-0.13 ± 0.20
	Advanced	1.1 ± 0.2^∗∗^	-0.10 ± 0.18	0.2 ± 0.1^∗∗∗^	0.13 ± 0.18	1.4 ± 0.2	0.03 ± 0.22
	Delayed	1.3 ± 0.1	-0.28 ± 0.18	0.1 ± 0.1^∗∗∗^	0.16 ± 0.18	1.2 ± 0.1	0.03 ± 0.20
	Drought	1.4 ± 0.1^∗^	-0.41 ± 0.18^∗^	0.1 ± 0.1^∗∗∗^	0.03 ± 0.18	1.1 ± 0.1	0.05 ± 0.21


*To visualize the results of the linear models, several regression lines for each treatment and each trait studied were drawn, based on significance for slopes and intercepts (**Figures 4**, **6**). Significance levels: ^∗∗∗^*p* < 0.001, ^∗∗^0.001 < *p* < 0.01, ^∗^0.01 < *p* < 0.05.*

### Specific Leaf Area

Across all species, only drought had an effect on SLA values; this effect was positive at low and negative at high altitudes (**Figure 3A**).

The growth rates of lowland species were significantly enhanced by drought along the whole altitudinal gradient, while upland species responded to this treatment negatively only at higher sites (**Figures 3B,C**, respectively). The general effect of snow manipulations on SLA was rather small; only SLA values of lowland species occurring at higher sites responded significantly positively to DE treatment.

Within the functional groups, SLA values of both forbs and legumes were significantly negatively affected by extreme drought (**Figures 4A,C**, respectively). In the latter case, the effect was detected only at higher altitudes.

Advanced snowmelt had a negative effect on SLA values of all PFTs studied, becoming progressively larger for graminoids occurring at higher altitudes and smaller for legumes growing at higher altitudes (**Figures 4B,C**, respectively). An altitudinal effect of AD treatment on SLA values of forbs was not detected (**Figure 4A**).

The effect of delayed snowmelt on SLA was found to be specific to PFT. Whereas only graminoids occurring at higher sites responded to this treatment (**Figure 4B**), SLA values of forbs, regardless of their location along the altitudinal gradient, were negatively affected by delayed snowmelt (**Figure 4A**). In the latter case, this effect was even stronger at higher altitudes. SLA values of legumes did not respond to this treatment (**Figure 4C**).

### Leaf Carbon Concentration

Among all species, only DE treatment positively affected LCC, being progressively larger at high altitudes (**Figure 3D**).

Apart from the negative effect of the DR treatment on upland species, none of the treatments had any remarkable effect on LCC of species with different altitudinal origin (**Figures 3D–F**).

Extreme drought, AD and DE treatments increased LCC in both graminoids and legumes, whereas no treatment effect in forbs was detected (**Figures 4D–F**).

### Leaf Nitrogen Concentration

Averaged over all species, LNC responded to all treatments; positively to delayed snowmelt and negatively to advanced snowmelt and drought (**Figure 3G**). In all cases, the effects were stronger at higher sites.

Apart from the positive effect of the DE treatment on species of upland origin occurring at high altitudes, none of the treatments had any remarkable effect on LNC of species with different altitudinal origin (**Figures 3H,I**, respectively).

Leaf nitrogen contents response to extreme drought was also found to be PFT specific. While trait values of forbs growing in higher altitudes decreased, graminoids exhibited a positive response to this treatment, becoming stronger at higher altitudes (**Figures 4G,I**, respectively). Any sign of the treatment having an effect on the LNC on legumes was not consistent along the altitudinal gradient, being positive in lower and negative in higher plots (**Figure 4I**).

Advanced snowmelt increased leaf nitrogen content in graminoids and legumes, where graminoids responded more strongly to this treatment at higher altitudes (**Figures 4H,I**). No effect of this treatment on leaf nitrogen in forbs was detected (**Figure 4G**).

Similarly, graminoids and legumes responded positively to the DE treatment showing a significant increase in LNC values (**Figures 4H,I**). Again, graminoids’ response to the treatment was stronger at higher altitudes. Forb LNC also increased under delayed snowmelt, however, this response was detected only at high altitudes (**Figure 4G**).

### Seed Number

Extreme drought negatively affected the seed number of all species, regardless their altitudinal distribution, being almost absent at higher altitudes (**Figures 5A–C**). In addition, AD treatment also significantly lowered the seed number of the upland species regardless of their location along the altitudinal gradient (**Figure 5C**).

The seed number of graminoids was increased by all the treatments (with a smaller effect from AD treatment on higher plots; **Figure 6B**), whereas forbs produced fewer seeds under extreme drought conditions (**Figure 6A**). Legumes profited from the AD treatment, producing a larger number of seeds (**Figure 6C**).

### Seed Mass

Across all species, only DR treatment negatively affected the seed mass of plants growing at high altitudes (**Figure 5D**). In contrast, extreme drought had a positive effect on the seed mass of upland species, being slightly smaller at high altitudes (**Figure 5F**). Seed mass of lowland species under the extreme drought was reduced (**Figure 5E**).

The response of lowland species to the AD treatment was two-part: in lower sites plants produced smaller seeds, whereas the seed mass in higher sites increased (**Figure 5E**). In contrast, seeds of the upland species were significantly larger under AD treatment regardless of their location along the altitudinal gradient (**Figure 5F**). Only the upland species responded to the DE treatment by producing larger seeds; this effect appeared to be consistent along the whole altitudinal gradient (**Figure 5F**).

Linear models showed that manipulative treatments had no significant effects on the seed mass of legumes (**Figure 6F**). Graminoid seed mass was reduced by both DE and DR treatments; these effects appeared to be consistent along the whole altitudinal gradient (**Figure 6E**). Seeds of the forbs were significantly smaller in AD treatment plots located at low altitudes and in DR treatment of higher plots. Furthermore, delayed snowmelt had a positive effect on seed mass of forbs, being progressively larger at high altitudes (**Figure 6D**).

## Discussion

There is an urgent need for knowledge about the potential effect of ECE on plant performance in general and for mountain plants in particular (Leingärtner et al., 2014; Hoiss et al., 2015). In the present study, we confirm previous findings that ECE considerably affect plant vegetative and generative traits (Jentsch and Beierkuhnlein, 2008; Jentsch et al., 2009). However, for the first time we show that the direction and magnitude of mountain plant performance responses to extreme drought, as well as advanced and delayed snowmelt depend on study site elevation, species’ altitudinal origin, and the PFT to which the species belongs.

### Overall Effects of the ECEs on Plant Performance

In mountain areas, the timing of the snowmelt determines the length of the growing season, which shortens with increasing altitude. Therefore, snow removal in the AD treatment significantly changed local temperature conditions in the treated plots, thus extending the vegetation period by ca. 18 days in lowland plots and by ca. 40 days at higher elevations. In contrast, temperature conditions in the DE plots were not much affected by the artificially increased snow layer, because the show melt date in this treatment was only 2–5 days later than on the control plots (Leingärtner et al., 2014). However, despite similar temperature conditions in the control plots, DE treatment had a positive effect on LCC and LNC of plants occurring in higher plots. Surprisingly, the AD treatment had a negative effect on LNC of plants occurring at higher elevations. Although this finding contradicts our assumption, we speculate that differences in soil fertility between the AD or DE treatments and control plots could account for the results. Indeed, in mountain systems, especially at high altitudes, soluble inorganic nitrogen (N) compounds and other N sources, such as dust particles and organic pollutants, stored in the snow pack represent a substantial additional source of N supporting plant growth early in the season (Bowman, 1992; Bilbrough et al., 2000; Hiltbrunner et al., 2005). Although not measured in our study, we assume that artificially increased snowpack enhanced N input in soils of the DE plots as compared to control plots. Since plants are capable of N uptake during snowmelt in alpine systems, regardless of their life form or functional type, plants occurring in higher plots may have acquired increased amount of snowmelt N through foliar or root uptake, once N entered the soil (Bilbrough et al., 2000). Therefore, the short-term improvement of the nutrient supply could stimulate usually N-limited photosynthesis and biomass accumulation in all of the species studied, especially in higher plots, which could lead to the observed increase in LNC and LCC [compare to Bowman et al. (1993) and Schäppi and Körner (1996)]. However, this increase of nutrients can be said to be relatively moderate, because it did not have any significant effect on the studied seed traits. Consequently, a lack of this important source of N explains the negative effect of the AD treatment on LNC as compared to control.

Extreme drought significantly lowered soil moisture in all plots equally along the whole altitudinal gradient (Leingärtner et al., 2014). Nevertheless, the apparent negative effect of this ECE on overall plant performance, namely smaller SLA values and lower LNC and smaller seeds, was present in upland sites only. These findings are in line with previous studies (Scott and Billings, 1964; Kuramoto and Bliss, 1970; Ehleringer and Miller, 1975; Enquist and Ebersole, 1994; Sardans et al., 2008) and are explained by extreme susceptibility of plants occurring at high elevation to water shortage. In the study region, high-elevation vegetation is adapted to high and regular levels of water supply in the form of higher precipitation rates and high air humidity (Körner, 1999; Gitlin et al., 2006; Ellenberg and Leuschner, 2010; Garcia-Fernandez et al., 2013). Therefore, water limitations during the relatively short high-altitude growing season significantly limited photosynthetic capacity and thus growth rates. Alternatively, these and other (see below) stronger effects on plant performance in higher elevations could be due to a methodological bias, namely the calculation of extreme drought duration for the study sites. As previously mentioned in the section ‘Materials and Methods’, climate data for lowland experimental sites (600–900 m a.s.l.) were used as the reference period to calculate a 1000-year extreme drought, because long-term meteorological observations in high altitudes (above 1000 m a.s.l.) of the study region are lacking. Since relative air humidity, as well as amount and frequency of summer precipitation, tend to increase with elevation (Körner, 1999; Ellenberg and Leuschner, 2010), it is likely that the calculation based on real high-altitude climatic data, would result in other value, putatively a shorter extreme drought. However, the direct soil water content measurements in the treatment plots indicated that the upland sites were not differently affected by the extreme drought (**Figure 2**) suggesting that the difference between the ‘real’ and calculated duration of extreme drought event is not that big.

### Effects of the ECEs on Trait Performance of Plants Originating from Different Elevations

#### Advanced Snowmelt

Despite a prolonged vegetation period, which is supposed to stimulate growth rates of lowland species (Woodward, 1975, 1979), AD treatment had no effects on the lowland species regardless of their altitudinal location. Growth traits of upland species also remained unaffected by this treatment. This conflict with our assumption that the performance of both lowland and upland species should be positively affected by the AD treatment may be explained by the fact that the temperature-induced increase in growth rates of fast-growing lowland species had to be supported by nutrients, in order to facilitate the allocation of newly produced photosynthetics to new tissue construction (Poorter and Remkes, 1990). However, resource-demanding lowland species occur in our study system on nutrient-poor soils (Rosbakh et al., 2015), thus they obviously took no advantage of the warmer temperatures of the advanced snowmelt. Furthermore, artificial snow removal in the AD treatment, especially in the higher plots, might also have significantly reduced soil N availability (see above), which in turn limited the growth rate of the lowland plants. Accordingly, low sensitivity of growth to enhanced temperatures as well as low nutrient requirements explain why none of the vegetative growth traits of plants belonging to this group were affected by AD treatment. Nevertheless, reduced soil N availability as the result of the artificial snowpack removal might still have had a negative effect on the reproductive traits of upland species, namely the reduction in seed number along with the increase in seed mass. Sexual reproduction (especially in high elevations), in contrast to photosynthetic vegetative growth that can be self-sustained, is strongly nutrient sensitive. Thus, when unfavorable environmental conditions necessitate a reduction in the reproductive output, plant species tend to alter seed mass at the expense of seed number, a trade-off between competitive ability and number of reproductive opportunities (Jakobsson and Eriksson, 2000). Consequently, the lower seed number of upland species in the AD treatment group provided an opportunity to allocate more resources into each seed, thus increasing the possibility of success under the assumed nitrogen scarcity because larger seeds provide more reserves for seedlings (Poorter et al., 1996; Pluess et al., 2005).

#### Delayed Snowmelt

In contrast to the AD treatment, vegetative growth trait responses of species with lowland and upland origin to the DE treatment differed due to their growth and nutrient acquisition potentials. Resource-demanding lowland species might benefit from the short-term improvement of nutrient supply (see above), as a result of the thicker snowpack in the treatment, resulting in enhanced growth rates (higher SLA values). Soil nutrients that are utilized, particularly N, were allocated to new tissue construction, which can be seen in the uniform values of LCC and LNC. Upland species also responded positively to the increasing N supply by increasing their leaf N concentrations with the strongest increase in higher sites. However, due to slower nutrient turnover, these species were not able to utilize the absorbed additional N for enhancing their growth rates (constant SLA values). In upland plants, especially growing on N-limited soils, stored N could be used to bridge asynchrony in nutrient supply and demand in certain environments, providing a form of insurance against catastrophe or sustaining the biochemical costs of reproduction [see Jaeger and Monson (1992) and references therein]. Therefore, larger seeds suggest that in our case absorbed nutrients were utilized to enhance usually N-limited reproduction in upland plants. This was probably achieved by releasing vegetative and reproductive growth from competition for N (Jaeger and Monson, 1992), allowing the plant to allocate more nutrients into seeds, thus increasing their chances of establishment in a nutrient-poor environment (Westoby et al., 1996; Pluess et al., 2005).

#### Drought

Vegetative traits of upland species were negatively affected by artificial drought (lower SLA values and lower LCC), whereas lowland species contrastingly responded positively (higher SLA values). The observed differences may be due to the fact that lowland and upland plant species are historically exposed to different water supply regimes. Historically, the performance of upland species in humid temperate zones such as the European Alps was mostly unconstrained by water shortage, because both precipitation and air humidity tend to be constantly high compared with lower altitudes (Körner, 1999). As a result, growth and reproduction of upland species is very susceptible to short periods of drought (Kuramoto and Bliss, 1970; Ehleringer and Miller, 1975; Enquist and Ebersole, 1994), even though soil water potential remains relatively high (Billings and Bliss, 1959). In contrast, the physiological optimum of lowland plants is under rather mesic and slightly dry conditions, because they generally occur in habitats with considerably less precipitation and lower air humidity (e.g., 1500 to 2600 mm precipitation per year in the study region; Marke et al., 2013) and 800 mm in Munich, which is located 140 km northwest from the study sites (Deutscher Wetterdienst^1^). Thus, this peculiarity allows lowland plants to occur in mountain regions not only by being resistant to drought, but also by benefitting from such conditions through enhancing their growth rates (i.e., higher SLA values). The higher resistance of lowland species to extreme drought was also confirmed by the lack of effects from the DR treatment on their reproductive traits.

Conversely, a lack of resistance to desiccation stress may explain the growth reduction of upland species. Despite apparent drought susceptibility, the individual seed mass of upland species increased in response to the DR treatment. This is in line with previous findings that plants tend to produce larger seeds under drought stress (Poorter et al., 1996), which may promote recruitment under dry conditions eventually repeated in the next or another growing season (Jakobsson and Eriksson, 2000). According to Galen (2000), larger seeds in drought-stressed plants are caused by the allocation trade-off between flower and seed mass, i.e., under water stress plants tend to have either small flowers that yield a heavy fruit set or large flowers with little to no fruit production, which may still serve to promote male fitness by providing pollen for other flowers.

### Effects of the ECEs on PFT Performance

Despite the fact that classification into PFTs proved to be largely unsatisfactory when generalizing responses and predicting effects (Dormann and Woodin, 2002; Parmesan and Hanley, 2015), some consistent differences in responses to the ECEs among PFTs were detected.

#### Advanced Snowmelt

Plant performance of both graminoids and legumes was positively affected by the AD treatment (higher LCC and LNC concentrations, and seed number), whereas forbs responded negatively to this treatment (lower SLA values and lower seed mass). Several reasons may explain the different responses of the PFTs to this treatment. We suggest that the improved temperature conditions of the extended growing season resulting from the AD stimulated photosynthetic rates of all studied PFTs. However, this enhancement in the form of a higher concentration of photosynthetics stored in tissues or higher growth rates can only occur if nutrient uptake is also enhanced (Tolvanen and Henry, 2001). It is possible that graminoids were able to adapt to the warmer conditions (higher LCC and LNC values) by taking up more nutrients to meet the needs of increased photosynthesis rates. This is due to either the inherited high N- and P-use efficiency (Bowman et al., 1995; Klanderud, 2008; Smilauerova and Smilauer, 2010), or their capability to occupy rapidly soil volume with available soil resources (Smilauerova and Smilauer, 2010). An extensive network of underground meristems that allows for a resource-sharing strategy (Zhang and Welker, 1996; Wellstein et al., 2017) could also contribute to the faster adjustment of graminoids to the AD treatment.

This opportunistic behavior of graminoids was not evident in the forbs. The opposite response for forbs (lower SLA values and lower seed mass) may have been due to the fact that their nutrient use is not as efficient (Bowman et al., 1995; Klanderud, 2008). Furthermore, their primary root system is only developed as a tap root (hence with relatively lower specific root area) with roots proliferating relatively slowly in nitrogen-enriched soil patches (Smilauerova and Smilauer, 2010). All the above could lead to the inability of forbs to support the increased physiological and photosynthetic activity by taking up more soil nutrients. Moreover, the putative reduction in soil nutrients, due to the artificial snow removal from the treatment area (see above), could also partially contribute to the conservative forb growth response to early snowmelt. In this context, the observed SLA reduction and seed mass in forbs could be explained by the lower soil N content.

Despite the fact that legume morphology and their capability of tracking environmental perturbations are similar to those of forbs, their performance was positively affected by the AD treatment (higher LCC and LNC values, higher number of produced seeds). We suggest that this is primarily due to the ability of legumes to forge a symbiotic N fixation that makes them less dependent on soil nutrient supply. For example, in the study of Jacot et al. (2000) it was found that, up to their altitudinal limit, legumes may acquire from 59 to 90% of their nitrogen through symbiotic N2 fixation depending on the species. Thus, thanks to this ability, legumes could meet the need for increased nutrients caused by the increased rates of photosynthesis that are reflected in higher photosynthetic concentrations in the leaves (higher LCC and LNC concentrations). Additionally, better performance of legumes (particularly higher foliar LNC) under snowmelt could be related to increased soil microbe activity, which is usually suppressed by low ambient temperatures (Schäppi and Körner, 1996; Kreyling, 2010).

It is also noteworthy that advanced snowmelt had, in general, no impact on growth rates (i.e., SLA) of either graminoids or legumes. We argue that extra photosynthetics (high LCC and LCN in legumes and graminoids) of plants exposed to the warmer environment of AD treatment were allocated to reproduction (higher seed number in both graminoids and legumes; **Figures 6B,C**) and not to new tissue construction, because nutrients strongly limit sexual reproduction in mountain ecosystems (Heer and Körner, 2002).

#### Delayed Snowmelt

Despite the relatively short length of the vegetation period, the majority of vegetative and reproductive traits of all PFTs studied responded positively to the DE treatment. Apparently, this positive treatment effect was not due to the changes in temperature, because the shortening of the vegetation season by less than 1 week would not have had any significant impact on the plants in the DE treatment. Taking into account the possible increase in N input from the artificially increased snowpack (see above) and considering morphological features (e.g., type of root system) along with physiological characteristics (e.g., N-use efficiency) of the PFTs studied, the observable pattern can be easily interpreted. After the snowmelt water entered the soil, the well-developed secondary root system of graminoids, spreading throughout the soil surface layers, could facilitate a more efficient uptake of additional nutrients, as compared to other PFTs, resulting in the stimulation of photosynthesis and growth (higher LCC and LNC, as well as SLA values). Although these two processes are nutrient limited in mountain ecosystems regardless of the PFT (Schäppi and Körner, 1996), graminoids usually have a greater response to N fertilization (Bowman et al., 1995), due to higher photosynthetic N-use efficiencies and higher photosynthetic rates than forbs and legumes on a per unit leaf area basis, and thus a greater potential supply of carbohydrates for growth (Bowman et al., 1995; Smilauerova and Smilauer, 2010). The nutrient amendment originating from the thicker snowpack also caused a shift in reproduction allocation patterns in graminoids in favor of higher seed number at the expense of their mass. This increases the chances of colonizing greater areas in the following spring (Jakobsson and Eriksson, 2000).

Increased primary productivity of graminoids may have enhanced their early-season competitive pressure on forbs (Zhang and Welker, 1996), resulting in reduced growth rates in this PFT (unaltered LCC and LNC, as well as lower SLA values), despite the putative N fertilization. Furthermore, we assume that increased competition strength of more responsive graminoids within the DE treatment area is responsible for the larger seeds in forbs, as compared to the control plots (**Figure 6D**). Larger seeds produce larger seedlings that therefore have an enhanced capacity to compete with increased graminoid dominance in the treated plot (Poorter et al., 1996; Jakobsson and Eriksson, 2000).

Another interesting finding of our study is that the intensity of the DE treatment effect on the performance of both graminoids and forbs increased with altitude. This could be either attributed to the increasing snowpack thickness (and therefore subsequent N input into soil) along the altitudinal gradient (52 cm at 641 m a.s.l. vs. 220 cm at 1984 m a.s.l.; Appendix 1) or the increasing dominance of graminoids with increasing altitudes. The abundance of graminoids in our study system gradually increased from 40 to 50% in the lower sites (mainly represented by *Arrhenatherum elatius*, *Helictotrichon pubescens*, and *Festuca rubra*) to up to 60–80% at subalpine-alpine sites, where *Carex sempervirens*, *C. firma* and *Sesleria albicans* dominate.

Although the morphological (mainly tap roots in lower soil layers) and physiological (comparatively low N- and water-use efficiency) characteristics of legumes are similar to those of forbs, their vegetative (higher LCC and LNC) and reproductive (higher number of produced seeds) traits responded positively to the DE treatment. This enhanced response is most likely due to the presence of N-fixing microorganisms which fix more nitrogen with an improved water supply (Gallacher and Sprent, 1978; Bilbrough et al., 2000; Lapointe, 2001). The additional nutrients taken up by legumes considerably stimulated their carbon assimilation (higher LCC and LNC as compared to control), however, due to their more conservative nature, the surplus assimilates were neither invested in new tissue production (stable SLA values) nor in sexual reproduction. We suggest that they were stored in leaves, to improve the legume’s fitness under the harsh growing conditions at high altitude. For example, the stored nitrogen and carbohydrates could bridge temporal gaps that exist between resource availability and resource demand in the upland environment, such as seasonal periods of cold and unpredictable gaps caused by calamities such as frost (Lapointe, 2001).

#### Drought

The simulation of an extreme drought significantly lowered soil moisture content in the DR plots (58% on average less than in the control), resulting in considerable changes in performance of the PFTs. However, plant performance under water stress was found to be PFT-specific. More specifically, the performance of graminoids was surprisingly positively affected by the DR treatment, showing a remarkable increase in all trait values (except for seed mass). Forbs responded to the extreme drought negatively (significant decrease in SLA and LNC, as well as seed number and seed mass). Legume response to the artificial drought was dual: SLA and LNC values were reduced, whereas LCC was higher in all plots along the whole altitudinal gradient as compared to the control. There are four possible explanations for this pattern. First and foremost the lack of a negative response from the graminoids to the extreme drought could be attributed to their root morphology, namely a comparatively higher specific root length and branching intensity, both of which are considered to be positively related to the efficiency of exploration and exploitation of mobile soil resources, such as water (Lapointe, 2001). In contrast, the coarse root systems of forbs and legumes could not sufficiently support increased water requirements, due to the extreme drought. Second, the increased performance of the graminoids in the DR treatment can be explained by their modular organization, which allows for a resource sharing strategy (Wellstein et al., 2017) that is lacking in forbs and legumes. In fact, there is evidence for a resource sharing of ramets (e.g., de Kroon et al., 1996; Alpert, 1999b) that even leads to overcompensation under drought (Alpert, 1999a). Third, the more competitive graminoids, as a consequence of their better adaptability to the extreme drought, could suppress the growth and reproduction of forbs and legumes (Smilauerova and Smilauer, 2010). Fourth, trait changes, for example, reduction in SLA, in the functional group of legumes most likely represent a phenotypic acclimation toward drought. Lower SLA is associated with a slower plant growth rate, which usually occurs under water stress and with enhanced water use efficiency under conditions of water stress [Wellstein et al. (2017) and citations therein]. Furthermore, increased leaf carbon in legumes confirms the adaptive nature of these changes; dehydration avoidance and tolerance mechanisms usually involve fructan maintenance and sucrose accumulation in plant organs exposed to drought (Zwicke et al., 2015). The only totally negative effect of the DR treatment on legume performance could be observed in the reduced LNC, particularly strongly in high elevations, which we relate to the negative effect of water shortage on symbiotic N-fixers [e.g., Sardans et al. (2008)].

Differences in the PFTs’ sensitivity to the extreme drought, due to the above-mentioned peculiarities, can also explain the contrasting responses of their reproductive traits to this treatment. Seed number increase along with seed mass decrease under conditions of water stress indicate an overall positive response of graminoid sexual reproduction to extreme drought. Production of a higher number of lighter seeds allows for an increase in colonization opportunities of this PFT (Jakobsson and Eriksson, 2000).

Like the vegetative traits, the response of forbs’ seed number and seed mass to the DR treatment was negative. However, it is difficult to assess whether the direct effect of the water deficits at the anthesis stage, which could induce flower sterility and reduce grain yield (Ekanayake et al., 1989), or reduced performance of mother plants (see above) were the causal basis for the observed reduced seed number and seed mass as compared to the control plots. In contrast, legumes displayed no changes in their seed reproduction output, probably, due to the better acclimation potential, as compared to forbs.

## Conclusion

Our data provide novel insights into the potential consequences of future climate change on the functioning of mountain plant communities. The results suggest that ECE can significantly modify the performance of specific plant groups and therefore lead to changes in plant community structure and composition under ongoing climate change. For instance, the immediate negative effects of extreme drought on the performance of susceptible upland species or forbs, especially at high elevations, could contribute to the process of thermophilization, a Europe-wide process of continuous advances of lowland plants into higher vegetation belts and shifts in effective physiological range of upland species toward summits as a result of recent warming (Jurasinski and Kreyling, 2007; Gottfried et al., 2012). Reduced growth rates, as well as low reproduction output of upland forbs, could, in the short term, provide more chances for lowland species (especially graminoids) to establish and/or increase their presence/dominance in plant communities. The increased presence of more competitive lowland species in alpine communities with short-stature, light-demanding and slow-growing cold-adapted species (Körner, 1999; Gottfried et al., 2012) could in turn lead to long-term changes in the upland community structure and composition, such as decreased/increased frequencies and abundancies of upland/lowland species and increased graminoid cover. In fact, this suggestion is supported by the data on long-term vegetation changes in the study region (Rosbakh et al., 2014). Our study therefore underlines the need for more experimental studies on the effects of ECE to understand the potential consequences of climate change for the alpine ecosystem.

## Author Contributions

BH, JK, and IS-D designed the study. SR, AL, and BH performed field work and analyzed data. SR wrote the first draft of the manuscript, and all authors contributed to revisions.

## Conflict of Interest Statement

The authors declare that the research was conducted in the absence of any commercial or financial relationships that could be construed as a potential conflict of interest.

## Footnotes

1 http://www.dwd.de
